# PD1^hi^ cells associate with clusters of proliferating B-cells in marginal zone lymphoma

**DOI:** 10.1186/s13000-018-0750-8

**Published:** 2018-09-15

**Authors:** Katherine Wickenden, Nadia Nawaz, Sami Mamand, Deevia Kotecha, Amy L. Wilson, Simon D. Wagner, Matthew J. Ahearne

**Affiliations:** Leicester Cancer Research Centre and Ernest and Helen Scott Haematology Research Institute, University of Leicester, Lancaster Road, Leicester, LE1 7HB UK

**Keywords:** Marginal zone lymphoma, Follicular helper T-cells, Spatial characteristics

## Abstract

**Background:**

Abnormally sustained immune reactions drive B-cell proliferation in some cases of marginal zone lymphoma but the CD4^+^ T-cell subsets, which are likely to contribute to the B-cell responses in the tumour microenvironment, are not well characterised and neither has the spatial distribution of the different subsets in involved lymph nodes been investigated.

**Methods:**

Employing a workflow of multiplex semi-automated immunohistochemistry combined with image processing we investigated association between infiltrating T-cells and proliferating lymphoma B-cells.

**Results:**

Both total numbers of activating follicular helper (Tfh) cells (defined by high expression of PD1) and suppressive regulatory (Treg) T-cells (defined by FOXP3^+^ expression) and the Tfh:Treg ratio, assessed over relatively large areas of tissue, varied among cases of marginal zone lymphoma. We determined spatial distribution and demonstrated that PD1^hi^ cells showed significantly more clustering than did FOXP3^+^. To investigate the association of infiltrating T-cells with lymphoma B-cells we employed Pearson correlation and Morisita-Horn index, statistical measures of interaction. We demonstrated that PD1^hi^ cells were associated with proliferating B-cells and confirmed this by nearest neighbour analysis.

**Conclusions:**

The unexpected architectural complexity of T-cell infiltration in marginal zone lymphoma, revealed in this study, further supports a key role for Tfh cells in driving proliferation of lymphoma B-cells. We demonstrate the feasibility of digital analysis of spatial architecture of T-cells within marginal zone lymphoma and future studies will be needed to determine the clinical importance of these observations.

## Background

Marginal zone lymphoma (MZL) includes three entities: nodal, extranodal (mucosa associated lymphoid tissue (MALT) lymphoma) and splenic marginal zone lymphoma (SMZL) [1]. These conditions share phenotypic and morphological features [1] but show varying genetic aberrations [2–5].

Extranodal MZL, the commonest of the three subtypes of MZL, has been linked to infectious micro-organisms [6] or autoimmune disorders prompting the idea that overactive immunity underlies lymphomagenesis [2] and this is supported by the association between some autoimmune conditions such as Sjögren’s syndrome with MZL.

There are recognised to be several different CD4^+^ T-cell subsets with different functions in normal immunity [7]. One of these subsets, follicular helper (Tfh cells) T-cells, is essential for normal immunity and is also required for the development of autoimmunity [8]. As well as characteristically producing IL-4 and IL-21, Tfh cells demonstrate high surface expression of PD1 (CD279) and nuclear expression of BCL6. Suppressive CD4^+^ T-cell subsets (regulatory T-cells (Tregs), (PD1^lo^ and FOXP3^+^), and follicular regulatory T-cells (Tfr) (PD1^hi^ and FOXP3^+^) counter the activating effects of Tfh cells [9].

Recent progress in computational biology that allows unbiased statistical modelling of the spatial distribution of lymphocytes has been applied to breast cancer in order to understand how the different cell types i.e. cancer cells, lymphocytes and stromal cells, interact with one another. This work has demonstrated that patterns of lymphocyte infiltration are prognostic [10] and specifically that Tfh cell infiltration and gene signature predicted response in breast cancer [11].

Numbers and pattern of T-cell infiltration have been demonstrated to correlate with some clinical characteristics in follicular lymphoma [12] and diffuse large B-cell lymphoma [13] and biologically this might be associated with their effects on B-cell proliferation: either activation (Tfh cells) or inhibition (Tregs). There are also recognised to be specific patterns of Treg infiltration in follicular lymphoma [12] but more detailed and quantitative investigation has been hampered because manual methods do not allow large areas of tissue to be analysed.

There has been much less work on T-cells in MZL. Tregs, are present in the tumor microenvironment (TME) in extranodal MZL [14] but activating follicular helper T-cells (Tfh) have not been characterised although they are a highly relevant subset because they are the principal producers of IL-21 and IL-4, which are growth factors important for normal and malignant B-cells [15]. MZL, unlike follicular lymphoma, usually has no discernible histological structure, which adds to the difficulty of detecting the spatial characteristics of infiltrating T-cells. In this report we combine immunohistochemistry and computational methods to show unexpected differences in the distribution of PD1^hi^ and FOXP3^+^ cells in MZL.

## Methods

### Samples

Fifteen MZL biopsy samples (spleen = 3, lymph node = 7, periorbital = 2, parotid, lung, thyroid = 1 each) were obtained from Leicester Royal Infirmary under Research Ethics Committee 14/EM/1176. (female = 11, male = 4; median age 63.5 years (range 48–74 years)). The characteristics of the patients and the treatment they received are shown (Table 1).

**Table 1:** Patient characteristics

Diagnosis		Histology	Age	Stage	LDH	TTFT	Alive	OS	Treatment
Extranodal	Periorbital	Diffuse	62	I	282	1	0	62	ISRT
Extranodal	Periorbital	Foliicular architecture with colonisation of follicles	59	I	257	NA	0	62	W&W
Nodal		Diffuse	67	IV	329	1	0	70	Rituximab+CHOP
Nodal		Diffuse	67	IV	424	1	0	49	Rituximab+FC
Extranodal	Periorbital	Residual germinal centres	48	IV	237	3	0	30	Rituximab+CVP + Rituximab maintenace
Nodal		Diffuse	61	III	227	5	0	36	Rituximab+CHOP
Nodal		Diffuse	73	I	248	NA	0	32	W&W
Nodal		Diffuse	73	IV	206	3	0	67	ISRT
Splenic		Diffuse	68	I	ND	NA	0	75	Splenectomy
Splenic		Diffuse	61	IV	ND	2	0	132	Splenic RT; CVP; Radiotherapy
Splenic		Diffuse	72	IV	290	2	1	1	Chlorambucil
Nodal		Diffuse	72	IV	277	1	1	1	Rituximab+CVP
Nodal		Diffuse	55	IV	234	9	0	62	Obinutuzumab+CVP + Obinutuzumab maintenance
Extranodal	Diffuse	Diffuse	74	I	ND	NA	0	31	Lung lobectomy
Extranodal	Diffuse	Diffuse	63	I	202	NA	0	25	W&W

The type of MZL is indicated (extranodal, nodal and splenic) and the site of extranodal disease together with the histological appearance (diffuse in 13/15 cases, with one case showing residual germinal centres and another case showing follicles with colonisation by lymphoma. Age (years), clinical stage (I to IV) and lactate dehydrogenase (LDH) are also shown. For LDH the upper limit of normal = 255 IU/L. ND not determined. Time to first treatment (TTFT) and overall survival (OS) in months is shown and whether the patients are alive (0) or dead (1). Three patients were managed by watch and wait (W&W) while the others received various treatments: involved site radiotherapy (ISRT), rituximab with cyclophosphamide, adriamycin, vincristine and prednisolone (CHOP), rituximab with cyclophosphamide, vincristine and prednisolone (CVP), obinutuzumab with CVP or rituximab with fludarabine and cyclophosphamide (FCR). Single agent chlorambucil, splenectomy or splenic radiotherapy were used as indicated

### Immunofluorescence microscopy

Immunofluorescence (IF) staining was performed using Opal 4-color fluorescent IHC kit (NEL800001KT, Perkin-Elmer, Waltham, MA, USA). Following antigen retrieval formalin fixed and paraffin embedded (FFPE) sections were incubated with protein block (RE7102, Novolink™ Polymer Detection System, Leica Biosystems, Wetzlar, Germany) for 10 min. For multiplex staining antibodies were used sequentially, first, anti-CD20 (1:100) (M0755, DAKO, Glostrup, Denmark), second anti-PD1 (used undiluted) (ab52587, Abcam, Cambridge, UK) and lastly anti-Ki67 (1:100) (M7240, DAKO) antibodies were incubated with tissue sections for 30 min at room temperature. Following incubation with HRP conjugated goat anti-mouse-IgG (1:200) (P0447, DAKO) for 1 h at room temperature slides were washed and incubated for 10 min at room temperature in the dark with Opal 520 (dilution 1:50) for anti-CD20, Opal 670 (1:100) for PD1 and Opal 570 (dilution 1:50) for Ki67. After additional washes nuclei were counterstained with DAPI (D1306, ThermoFisher). A negative control slide, incubated with mouse IgG1 (X0931, DAKO) was included in each staining run. Images of the stained slides were recorded using NanoZoomer-XR Digital slide scanner C12000–01 (Hamamatsu Photonics, Hamamatsu, Japan) and the images were pseudo-coloured and merged by using ImageJ analysis software [16].

### Immunohistochemistry

**Fig. 1:**
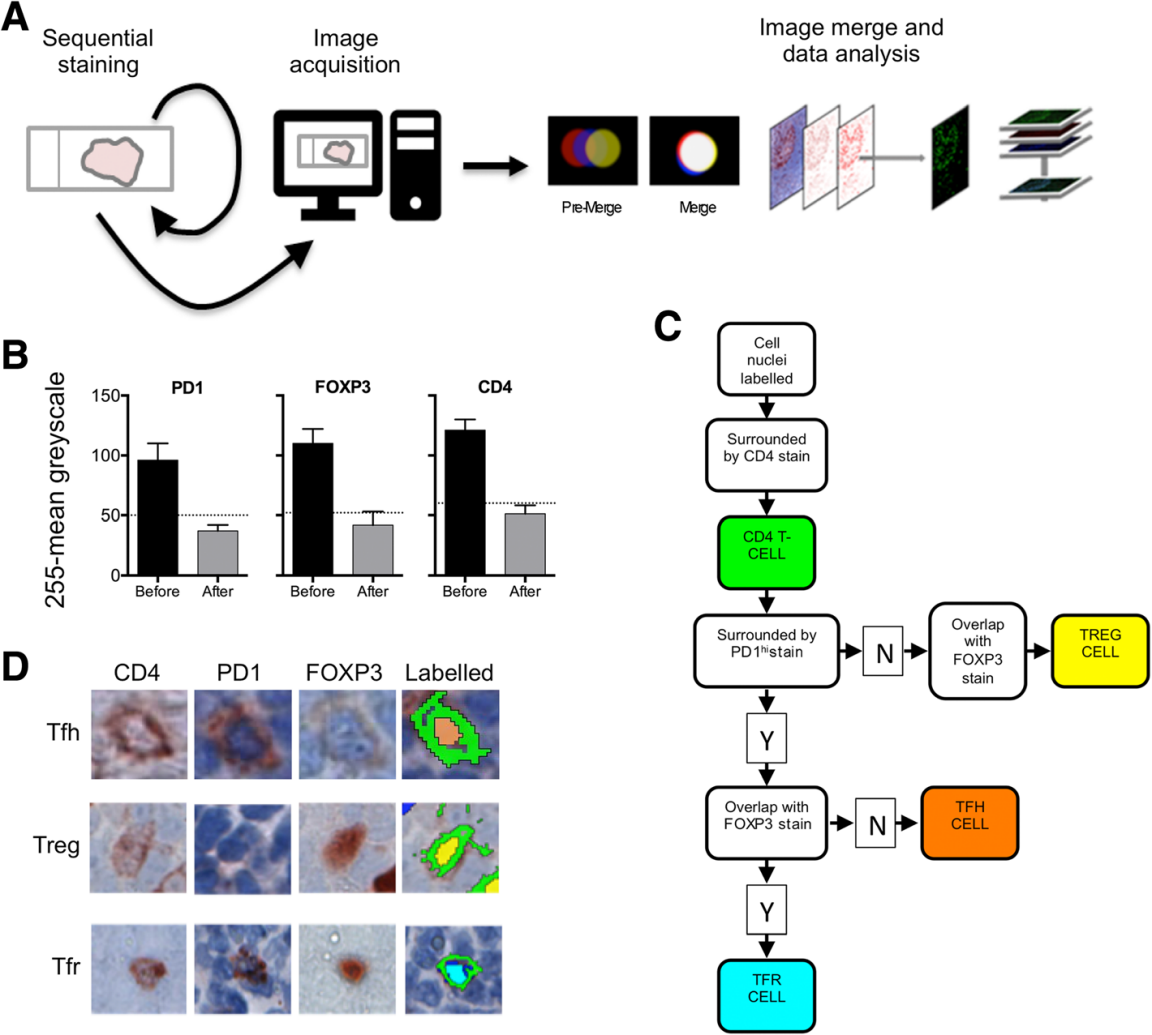
Workflow for immunohistochemistry and image analysis. **a** Diagram of the immunohistochemical workflow. **b** Quantification of the efficiency of elution for three antibodies: anti-PD1, anti-FOXP3 and anti-CD4. Black bars represent antibody staining intensity (mean ± SEM) and gray bars represent staining intensity following elution. Each experiment was carried out three times. The dotted line is the background intensity produced by an unstained tissue section. **c** Flow diagram to show Visiopharm methodology (www.visiopharm.com) used to analyse tissue sections stained sequentially with anti-CD4, anti-PD1 and anti-FOXP3 antibodies. The algorithm first identified cell nuclei by haematoxylin counterstain following which CD4 expressing cells were marked green. Next CD4 expressing cells were interrogated for co-expression of FOXP3. There was then a branch point in the algorithm. If FOXP3 expression was detected but there was no co-expression of PD1 at a high level (PD1^hi^), as previously defined by comparison with tonsillar Tfh cells, then the cell was determined to be Treg and the nucleus was labelled yellow. However, if the cell co-expressed CD4, FOXP3 and was PD1^hi^ then it was determined to be a Tfr cell and the nucleus was labelled turquoise. Cells that were CD4 expressing and PD1^hi^, but not FOXP3 expressing were determined to be Tfh cells and the nuclei were labelled orange. Cells of each subset were then counted digitally. **d** Identification of specific T-cell subsets by multiplex immunohistochemistry. Tissue sections were stained with anti-CD4, anti-PD1 (EuroMabNet, clone NAT105) and anti-FOXP3 (EuroMabNet, clone 236A). Example Tfh cell (CD4^+^PD1^+^FOXP3^−^) painted orange, Treg cell (CD4^+^PD1^−^FOXP3^+^) painted yellow and Tfr cell (CD4^+^PD1^+^FOXP3^+^) painted turquoise are presented

In order to identify specific T-cell subsets by multiplex immunohistochemistry (workflow illustrated in Fig. 1a) FFPE tissue sections were stained with anti-CD4 (4B12, DAKO) (1:80 dilution for 20 min), anti-PD1 (EuroMabNet, clone NAT105) (used undiluted for 60 min) and anti-FOXP3 (EuroMabNet, clone 236A) (1:100 dilution for 60 min) [17, 18]. For all antibodies antigen retrieval was with Tris-EDTA pH 9. Peroxidase-coupled secondary antibody (MP-7452, Vector Laboratories, Burlingame, CA, USA) was employed with AEC-peroxidase substrate (SK-4205, Vector Laboratories). Tissue sections were processed through sequential staining and destaining (glycine (25 mM), 1% SDS, pH 2) following which image data was acquired and downstream processing carried out. This involved registration of the separately stained images followed by data analysis, which includes both enumeration of stained cells and also spatial information i.e. co-ordinates of each cell (Visiopharm, Hoersholm, Denmark). All images that were taken forward for data analysis were carefully checked for successful registration. Persisting antibody from early rounds of staining could be a confounding factor and we, therefore, confirmed that the elution process reduced residual staining to background levels (Fig. 1b). Visiopharm apps were developed (available on application to the authors) to paint Tfh cells (CD4^+^PD1^hi^FOXP3^−^) orange, Treg cells (CD4^+^PD1^−^FOXP3^+^) yellow and Tfr cells (CD4^+^PD1^hi^FOXP3^+^) turquoise (Fig. 1c and d) prior to extracting cell numbers and location coordinates. An intensity threshold for the definition of high PD1 expression was set by utilising PD1 expression of Tfh cells within tonsillar germinal centres from healthy subjects.

**Fig. 2:**
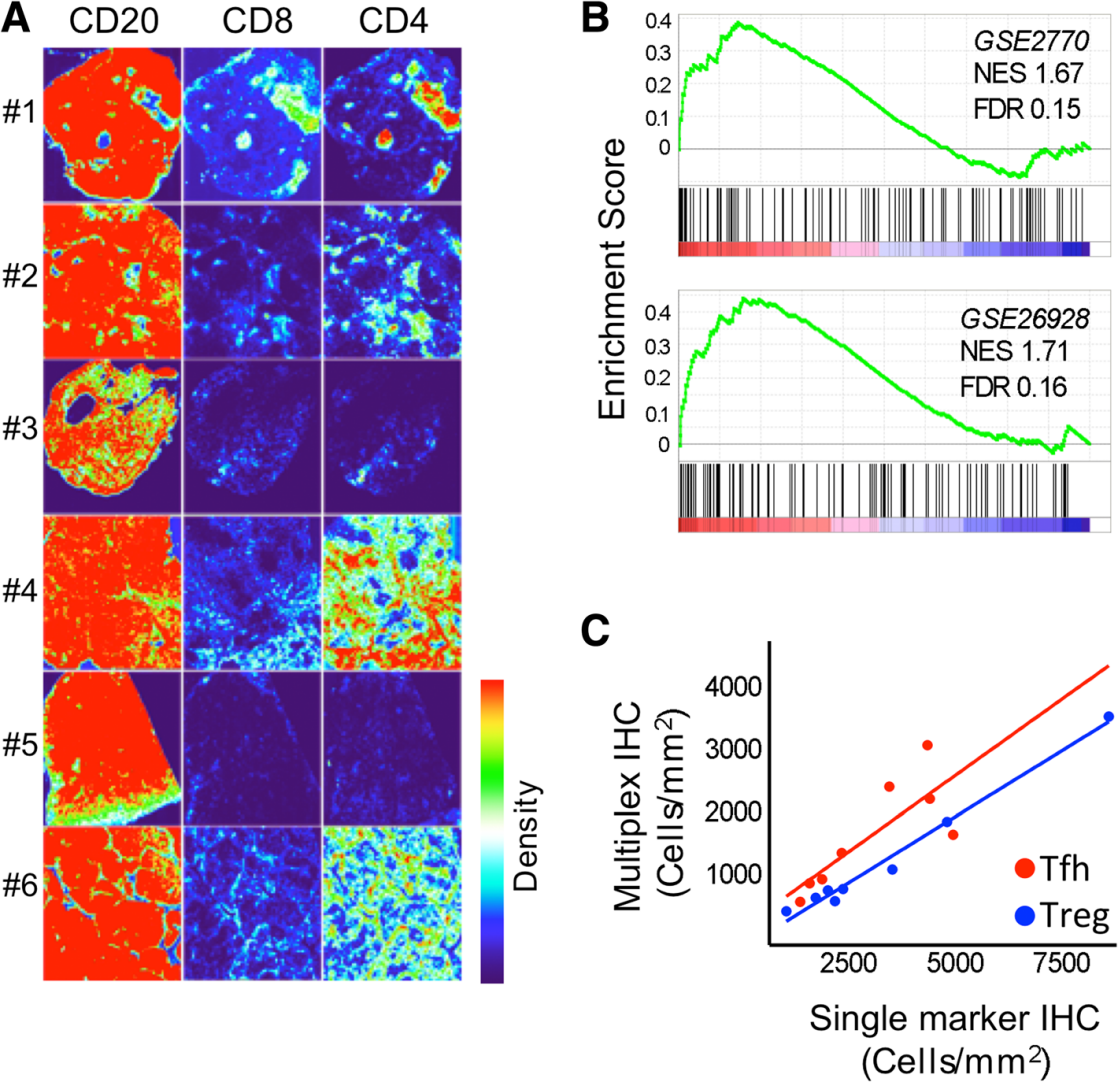
Architecture of CD4+ T-cell infiltration and T-cell gene signatures. **a** Tissue sections from 6 cases were stained with anti-CD20, anti-CD4 and anti-CD8. Surface plots generated in ImageJ (https://imagej.net/Fiji) are shown for each stain. Intensity has been normalised so that images can be compared. T-cell zones are evident and whilst these comprised both CD4 and CD8 T-cells, CD4 T-cells were found at higher density in 4 of 6 cases (#1, #2, #4 and #6). **b** Gene set enrichment analysis. Data from Arribas et al. (2012)^5^ was employed to discover T-cell signatures. Utilising a false discovery rate (FDR < 0.25) for exploratory work. Two gene signatures were found to be positively enriched in MZL. NES is normalised enrichment score. **c** Support for use of PD1^hi^ and FOXP3^+^ as single surrogate markers of Tfh and Treg cells respectively. Numbers of Tfh and Treg cells, as defined (Fig. 1d), were compared against single marker expression of PD1^hi^ and FOXP3

Tissue sections from 6 cases (Fig. 2b) were stained with anti-CD20 (M0755, DAKO), anti-CD4 (M7310, DAKO), and anti-CD8 (M7103, DAKO). Surface plots generated in ImageJ (https://imagej.net/Fiji) are shown for each stain. Intensity has been normalised so that images can be compared.

### Statistics

Distance from either FOXP3^+^ cells or PD1^hi^ cells to the nearest Ki-67 cell was plotted for 10^5^ cells sampled from 10 cases. All clustering and spatial analysis was performed using the statistical programme R (Spatstat package [19] and ggplot2 package [20]).

R was also used to determine Ripleys K function, an analysis tool to quantify the spatial distribution observed for each cell subtype as compared to complete spatial randomness (CSR).

Other statistical tests (Mann-Whitney, Kruskal Wallis tests and linear regression) were carried out with Prism 6 software (GraphPad Software, La Jolla, CA). Gene set enrichment analysis [21] (http://software.broadinstitute.org/gsea/index.jsp) was employed to analyse gene expression data from a publicly available database [22] under accession numbers GSE32233 and GSE32231.

## Results

### T-cell infiltration and T-cell gene expression signatures

To gain an understanding of T-cell distribution in MZL multiplex semi-automated immunohistochemistry was carried out for CD20, CD4 and CD8. Whole tissue heat-maps show the density of infiltrating T-cells (Fig. 2a). These reveal T-cell zones comprising low numbers of CD8^+^ and abundant CD4^+^ T-cells with clearly differing patterns of spatial localisation.

To provide support for the presence of activated T-cells in the MZL TME we carried out gene set enrichment analysis on data from nodal MZL [22]. Two signatures (GSE2770 - genes up-regulated in CD4^+^ lymphocytes induced to differentiate into Th1 and Th2 by treatment with IL-12 and IL-4 respectively in the presence of TGFbeta and GSE26928 - genes up-regulated in effector memory T-cells as compared to CXCR5^+^ T-cells) of polarised CD4^+^ T-cells (Fig. 2b) were obtained (FDR < 0.25). The tissue density maps and GSEA together suggest effector CD4^+^ T-cells are present in the tumour microenvironment.

### Quantitation of CD4^+^ T-cell subsets

Next we used image analysis to overcome biased representation due to counting relatively small fields and acquired similar quantitative information about infiltrating CD4^+^ T-cell subsets (Tfh, Tfr and Treg).

We defined Tfh cells as CD4^+^PD1^hi^FOXP3^−^, Tfr cells as CD4^+^PD1^hi^FOXP3^+^, and Treg cells as CD4^+^PD1^−^FOXP3^+^ (Fig. 1c and d). Collectively these three populations accounted for 16.4 ± 3.6% mean ± SEM) of total CD4^+^ cells (*n* = 8). Tfr cells represented only a rare population accounting for 4.6 ± 1.3% (mean ± SEM) of CD4^+^PD1^hi^ and were not anlaysed further. We found strong associations between PD1^hi^ and Tfh cell numbers (R^2^ = 0.98; *P* < 0.0001) and FOXP3^+^ and Treg numbers (R^2^ = 0.64; *P* = 0.017). Therefore, to facilitate this analysis and future translational lymphoma research we followed others in employing single markers, either PD1^hi^ or FOXP3^+^ [12, 23] as surrogates for Tfh and Treg subsets respectively (Fig. 2c).

**Fig. 3:**
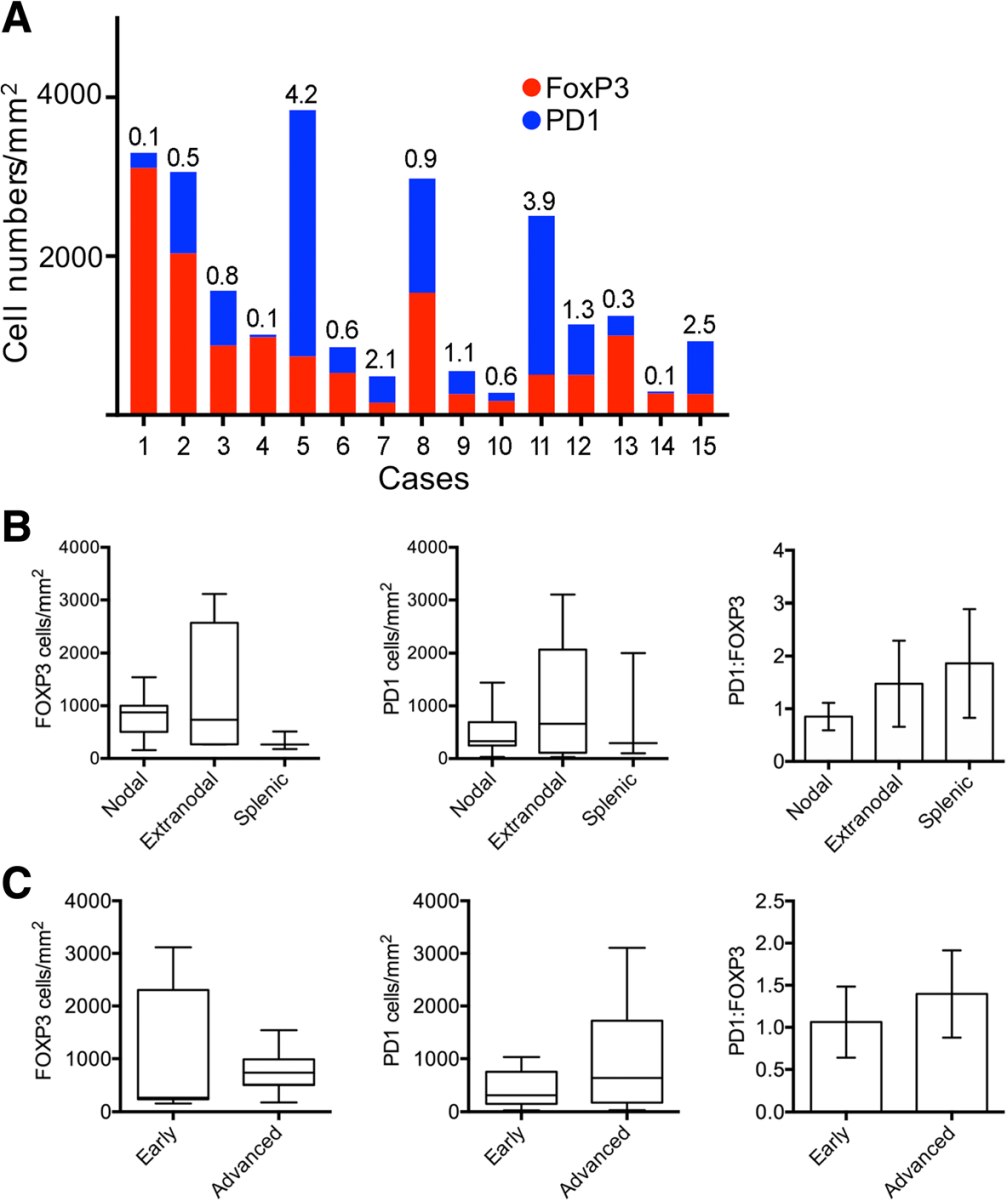
Automated quantitation of marker proteins. **a** Automated quantitation of PD1^hi^ and FOXP3^+^ cells in 15 cases of MZL. Numbers above bars represent PD1^hi^FOXP3^+^ ratio. **b** Analysis of cell density data and PD1^hi^:FOXP3^+^ ratio by type of MZL: nodal, extranodal or splenic. Box and whisker plots show median, 90th and 10th centile. **c** Analysis of cell density data and PD1^hi^:FOXP3^+^ ratio by stage of disease, either early stage (I and II) or advanced stage (III and IV)

Total numbers of PD1^hi^ and FOXP3^+^ numbers varied over ~ 20-fold with some cases having very much heavier T-cell infiltration than others (Fig. 3a). The ratio (PD1^hi^:FOXP3^+^) also varied with activating PD1^hi^ cells the majority in some cases whereas suppressive FOXP3^+^ cells were dominant in others.

Analysing SMZL, nodal and extranodal MZL separately showed no significant differences between overall density of PD1^hi^ or FOXP3^+^ cells or PD1^hi^:FOXP3^+^ in the three groups (Kruskal Wallis test) (Fig. 3b). There were also no significant differences in T-cell density or PD1^hi^:FOXP3^+^ when the cases were analysed by stage (Fig. 3c).

### Spatial distribution of T-cells

**Fig. 4:**
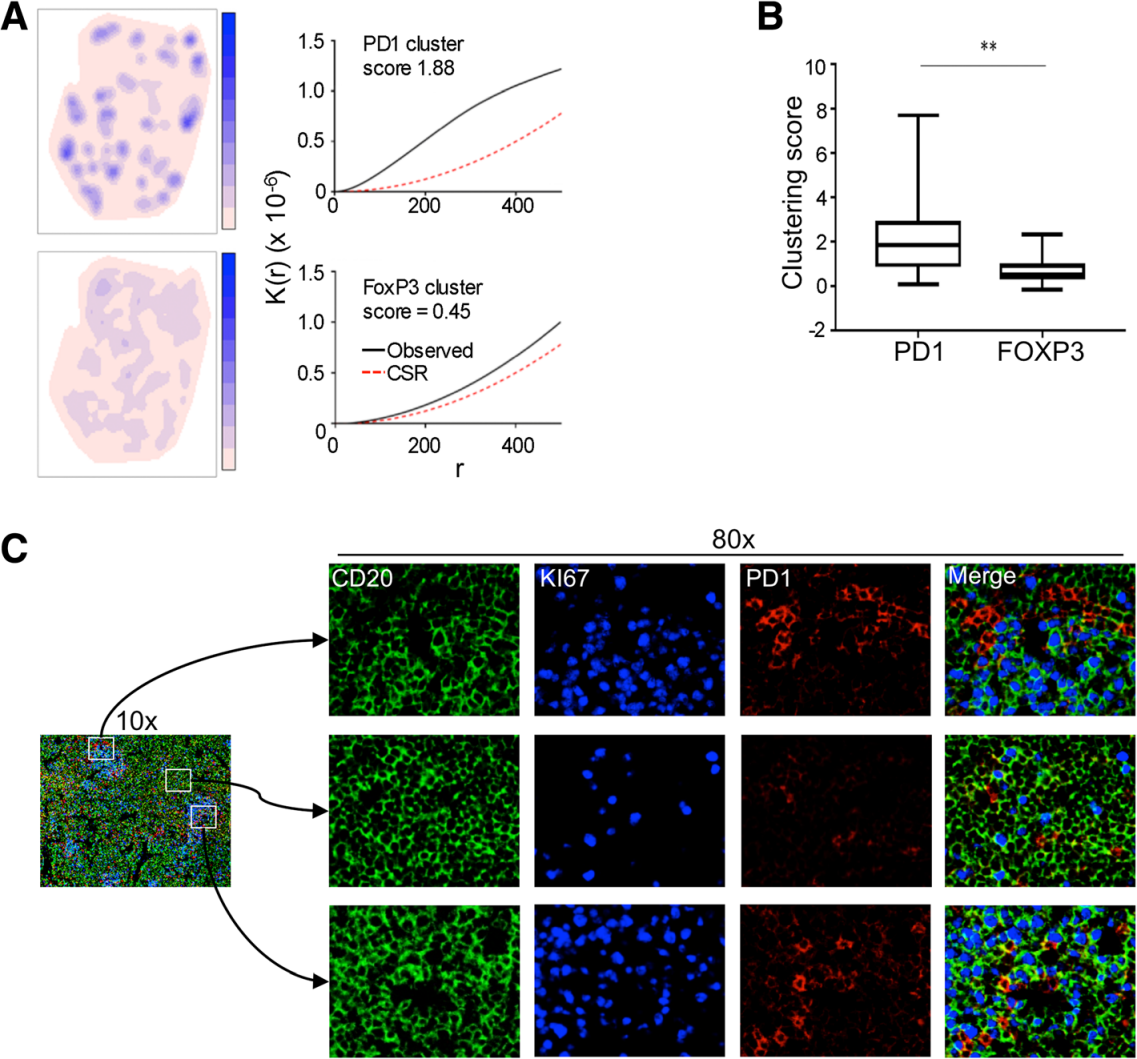
Clustering of PD1^hi^ cells. **a** Left hand panels are density plots representing PD1^hi^ (top) and FOXP3 (bottom) for one case (lymph node tissue). The impression of greater clustering of PD1^hi^ cells is confirmed by statistical analysis using Ripleys K function (right hand panels). A measure of distance (r) on the x-axis is plotted against K(r) function on the y-axis. The curve for complete spatial randomness (CSR) (dotted red line) is compared to K function for MZL (solid black line) for PD1 (upper panel) and FOXP3 (lower panel). Greater upward deflection of the observed curve compared to CSR represents greater clustering with the difference between these curves calculated to provide an overall cluster score. **b** Cluster scores for MZL cases (*n* = 15). PD1^hi^ cells are significantly more clustered than FOXP3^+^ cells (Mann-Whitney test, *P* < 0.01). **c** Immunofluorescence images. CD20 (green), Ki-67 (blue) and PD1 (red) staining of a lymph node from a patient with MZL. Low power (10×) views of the merged antibody stains show that proliferating cells occur in clusters. Three fields are shown as high power views (80×). Two of these fields are from regions that contain a high density of Ki-67^+^ cells (upper and lower rows) while the middle row is from a region of lower Ki-67^+^ density

Next, we chose to explore the spatial distribution of infiltrating PD1^hi^ and FOXP3^+^ cells, having observed various patterns of CD4^+^ T-cell infiltration (Fig. 2a), in order to determine whether these cells are randomly distributed or clustered. Ripley’s K test, was applied to data from whole tissue sections (mean area 50.7mm^2^, range 4.8–112 mm^2^) (*n* = 15) (Fig. 4a). Overall we found that PD1^hi^ cells were significantly more clustered i.e. greater separation of observed curve (solid black lines in Fig. 3a) from CSR (dotted red lines), than FOXP3^+^ cells (median cluster score 1.84 for PD1^hi^ cells and 0.45 for FOXP3^+^ cells; Mann-Whitney test *P* = 0.003) (Fig. 4b).

**Fig. 5:**
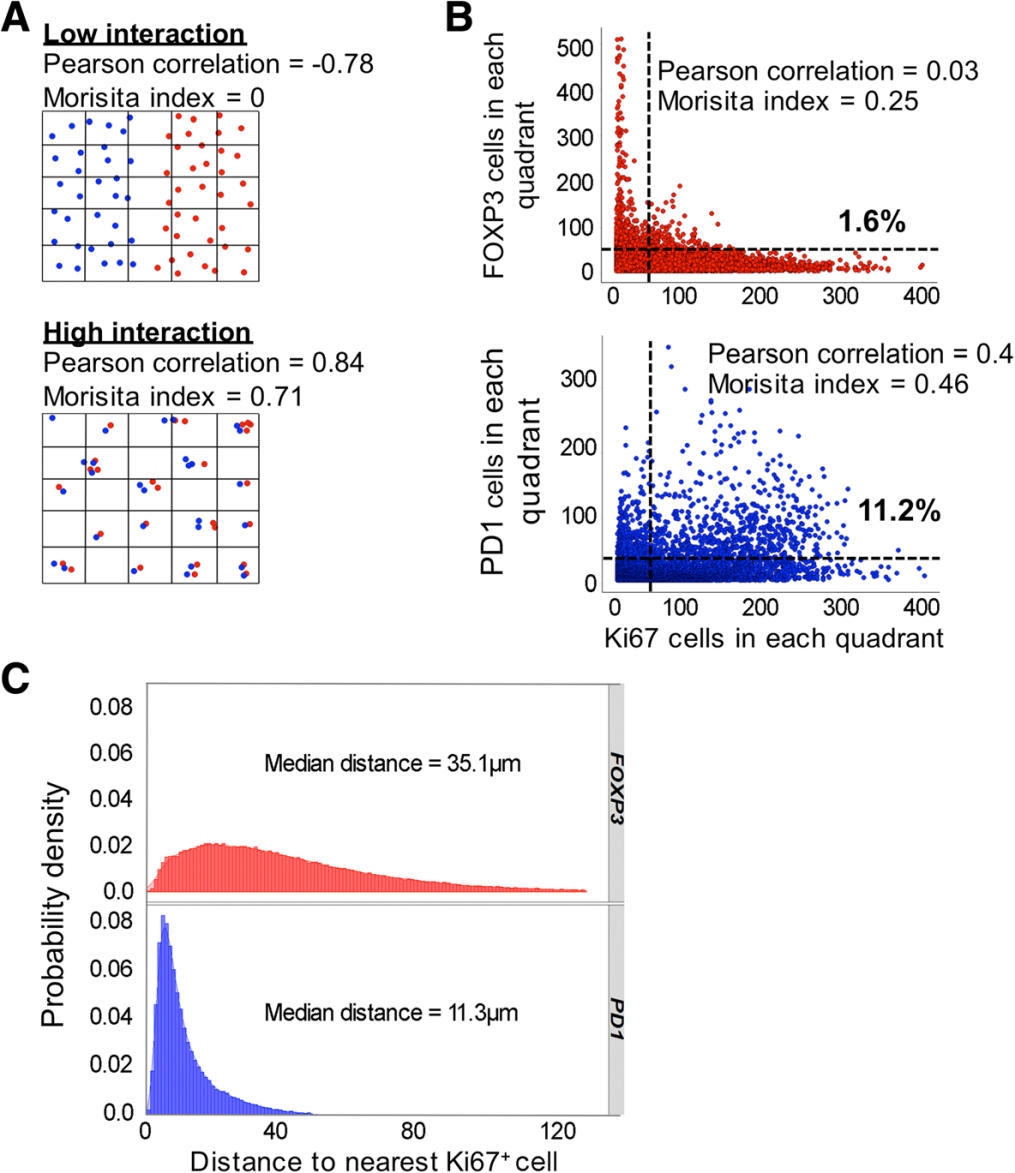
PD1^hi^ cells cluster with Ki-67^+^ cells. **a** Illustrative cartoon depicting low and high interactions with the respective Pearson correlation and Morisita-Horn index for both patterns. **b** Spatial interaction between FOXP3 or PD1 and Ki-67 cells. Scatter plots displaying Ki67^+^ against PD1^hi^ cells (upper panel) and FOXP3^+^ cells (lower panel) measured in spatial quadrants (200μm^2^). Only quadrants containing at least 5 cells of any population are displayed. Quadrants containing higher densities of both cell types (> 50 cells) were considered to represent high interaction. 11.3% of all quadrants analysed for Ki67^+^ and PD1^hi^ cells met this definition of high interaction compared to only 1.6% of Ki67^+^ and FOXP3^+^ cells. Analysis was repeated for different sized quadrants (100 and 400μm^2^) confirming similar results. **c** Nearest neighbour analysis. Distance from either FOXP3^+^ cells (upper panel) or PD1^hi^ cells (lower panel) was plotted for 10^5^ cells sampled from 10 cases. For PD1^hi^ cells the large peak occurring at short distances to the nearest Ki-67^+^ cell indicates that these cells are closer to Ki-67^+^ cells than FOXP3^+^ cells, which are more evenly distributed. All clustering and spatial analysis was performed using the statistical programme R

Tfh cells strongly drive B-cell proliferation and even extra-nodal CD4^+^PD1^hi^ T-cells are able to secrete factors enabling B-cell help [24]. We hypothesised that the PD1^hi^ cells might associate with proliferating lymphoma B-cells. To support this idea we first carried out immunofluorescence microscopy on a single case (Fig. 4c). This showed that proliferating Ki-67^+^ cells were predominantly B-cells and formed clusters but without the structure of normal or residual germinal centres. There was also an apparent association between areas with a high density of proliferating cells and PD1^hi^ cells. In order to support the hypothesis that PD1^hi^ cells associate with Ki-67^+^ lymphoma B-cells we quantified the spatial interaction between Ki67^+^ cells and Ki67^−^PD1^hi^ T-cells. Cell co-localization was objectively determined by virtually tessellating stained tissue sections into non-overlapping equally sized quadrants prior to cell counting within each defined spatial area [25]. Two measures, Pearson correlation and Morisita-Horn index, an ecological measure of interaction (illustrative examples Fig. 5a), were employed to analyse data from 17,000 200μm^2^ quadrants from 10 patients. Ki67^+^ and PD1^hi^ cells demonstrated closer interaction than did Ki67^+^ and FOXP3^+^ cells (Pearson correlation 0.4 vs 0.03; Morisita-Horn index 0.46 vs 0.25, Fig. 5b). To further interrogate spatial interaction we compared the distance between PD1^hi^ or FOXP3^+^ cells and the nearest Ki67^+^ cell in a nearest neighbour analysis. Ki67^−^PD1^hi^ cells (median distance 11.3 μm) were significantly more likely to be found in close proximity to Ki67^+^ B-cells than were Ki67^−^FOXP3^+^ cells (median distance 35.1 μm) (Mann-Whitney test *P =* < 0.0001) (Fig. 5c) suggesting for the first time that infiltrating Tfh cells might have a role in driving proliferation, and hence contribute to the varying clinical course of MZL.

## Discussion

In this report we demonstrate unexpected architectural complexity within MZL, defined by the analysis of single surface markers, PD1, representing Tfh cells and FOXP3, representing Tregs. Infiltrating CD4^+^ T-cell subsets have been studied by others in follicular lymphoma and the distribution of cells in relation to the follicle associates with clinical characteristics [26]. MZL shows histological heterogeneity: while some cases show a diffuse pattern of infiltration in others a follicular pattern is observed. Residual germinal centres, which are presumed to be normal reactive follicles, are also a feature of MZL. Residual germinal centres show the characteristic features of a polarised structure with BCL6 expressing proliferating B-cells in the dark zone and Tfh cells within the light zone. A limitation of our study is that we cannot entirely resolve the issue of whether the Tfh clusters we observe represent residual germinal centres. Inspection of the PD1 stained slides suggested that, in our series there were relatively few residual germinal centres and, therefore, our data supports the notion that Tfh clusters are a feature of the lymphoma but does not definitively exclude the possibility that some clusters are due to residual germinal centres. This study also prompts further questions about possible extra layers of complexity in the structure of MZL, which lie outside the scope of this work. For example, could Tfh clusters be associated with residual germinal centres?

In order to determine whether patterns of cell distribution are non-random analysis has to be carried out across whole tissue sections using an unbiased biostatistical approach. The number of patient cases in this study is low and we cannot, therefore, make definitive statements about the spatial characteristics of infiltrating T-cells in a disease as diverse as MZL. However, using automated techniques we determined spatial characteristics over large areas and taking into account data from 10^6^ cells (Fig. 5c) this allows robust and statistically significant conclusions to be drawn and, in turn, this allows us to generate hypotheses about the role of infiltrating T-cells in MZL.

CD4^+^ T-cell subsets are characterised by multiple surface markers, cytokine production and transcription factors. Supported by the work of others [23] who demonstrated that PD1 was highly expressed only on Tfh cells in follicular lymphoma we have used PD1^hi^ as a single marker to define Tfh cells. Both Treg and Tfr populations express FOXP3. We are interested in translating our work into the clinical laboratory and, therefore, we chose to employ immunohistochemistry for the bulk of the work described here although our observation of possible interaction between PD1^hi^ cells and B-cells was made by immunofluorescence microscopy.

We showed good correlation between single marker and multiplex immunohistochemistry (Fig. 2c) and chose to employ single markers because the simplicity of this methodology will facilitate future studies involving larger numbers of patients. We show the feasibility of mapping the spatial architecture of T-cell infiltration in MZL but the clinical impact of our findings in MZL, and possibly other low-grade B-cell lymphoma subtypes, needs testing prospectively. Importantly, we suggest the hypothesis that Tfh cells have a role in driving B-cell proliferation in MZL. This idea is compatible with the biological role of normal Tfh cells, which are required for driving B-cell proliferation [27] and antibody production [28] and also supports evidence for Tfh involvement in follicular lymphoma [29, 30].

## Conclusions

Our data suggests that CD4^+^ T-cell subsets other than Tfh cells are present in the tumour microenvironment of MZL warranting further studies characterising these and establishing their biological and clinical significance. The mechanisms determining tumour ecology in MZL are unclear but a testable hypothesis is that the specific mutational spectrum of the B-cell lymphoma influences the character of T-cell infiltration in the TME. Overall combining spatial characterisation and genomic features, such as IgVH repertoire and KLF2 mutational status [31], might identify MZL patients with poor outcomes and help to define effective treatments.

## Abbreviations

CSR: Complete spatial randomness
MZL: Marginal zone lymphoma
Tfh: Follicular helper T-cell
Tfr: Follicular regulatory T-cell
TME: Tumor microenvironment
Treg: Regulatory T-cell

## References

[1] Zinzani PL (2012). The many faces of marginal zone lymphoma. Hematology Am Soc Hematol Educ Program.

[2] Du M-Q (2017). MALT lymphoma: genetic abnormalities, immunological stimulation and molecular mechanism. Best Pract Res Clin Haematol.

[3] Parry M., Rose-Zerilli M. J. J., Ljungstrom V., Gibson J., Wang J., Walewska R., Parker H., Parker A., Davis Z., Gardiner A., McIver-Brown N., Kalpadakis C., Xochelli A., Anagnostopoulos A., Fazi C., Gonzalez de Castro D., Dearden C., Pratt G., Rosenquist R., Ashton-Key M., Forconi F., Collins A., Ghia P., Matutes E., Pangalis G., Stamatopoulos K., Oscier D., Strefford J. C. (2015). Genetics and Prognostication in Splenic Marginal Zone Lymphoma: Revelations from Deep Sequencing. Clinical Cancer Research.

[4] Rinaldi A, Mian M, Chigrinova E, Arcaini L, Bhagat G, Novak U (2011). Genome-wide DNA profiling of marginal zone lymphomas identifies subtype-specific lesions with an impact on the clinical outcome. Blood.

[5] Arcaini L, Rossi D, Paulli M (2016). Splenic marginal zone lymphoma: from genetics to management. Blood.

[6] Zucca E, Bertoni F, Vannata B, Cavalli F (2014). Emerging role of infectious etiologies in the pathogenesis of marginal zone B-cell lymphomas. Clin Cancer Res.

[7] Fang D, Zhu J (2017). Dynamic balance between master transcription factors determines the fates and functions of CD4 T cell and innate lymphoid cell subsets. J Exp Med.

[8] Vinuesa CG, Linterman MA, Yu D, Maclennan ICM (2016). Follicular helper T cells. Annu Rev Immunol.

[9] Linterman MA, Pierson W, Lee SK, Kallies A, Kawamoto S, Rayner TF (2011). Foxp3+ follicular regulatory T cells control the germinal center response. Nat Med.

[10] Nawaz Sidra, Yuan Yinyin (2016). Computational pathology: Exploring the spatial dimension of tumor ecology. Cancer Letters.

[11] Gu-Trantien C, Loi S, Garaud S, Equeter C, Libin M, de Wind A (2013). CD4^+^ follicular helper T cell infiltration predicts breast cancer survival. J Clin Invest.

[12] Carreras J, López-Guillermo A, Fox BC, Colomo L, Martinez A, Roncador G (2006). High numbers of tumor-infiltrating FOXP3-positive regulatory T cells are associated with improved overall survival in follicular lymphoma. Blood.

[13] Ahearne MJ, Bhuller K, Hew R, Ibrahim H, Naresh K, Wagner SD (2014). Expression of PD-1 (CD279) and FoxP3 in diffuse large B-cell lymphoma. Virchows Arch.

[14] Craig VJ, Cogliatti SB, Arnold I, Gerke C, Balandat J-E, Wündisch T (2010). B-cell receptor signaling and CD40 ligand-independent T cell help cooperate in helicobacter-induced MALT lymphomagenesis. Leukemia.

[15] Ahearne MJ, Willimott S, Piñon L, Kennedy DB, Miall F, Dyer MJS (2013). Enhancement of CD154/IL4 proliferation by the T follicular helper (Tfh) cytokine, IL21 and increased numbers of circulating cells resembling Tfh cells in chronic lymphocytic leukaemia. Br J Haematol.

[16] Schneider CA, Rasband WS, Eliceiri KW (2012). NIH image to ImageJ: 25 years of image analysis. Nat Methods.

[17] Glass G, Papin JA, Mandell JW (2009). SIMPLE: a sequential immunoperoxidase labeling and erasing method. J Histochem Cytochem.

[18] Pirici D, Mogoanta L, Kumar-Singh S, Pirici I, Margaritescu C, Simionescu C (2009). Antibody elution method for multiple immunohistochemistry on primary antibodies raised in the same species and of the same subtype. J Histochem Cytochem.

[19] Baddeley A, Rubak E, Turner R (2015). Spatial point patterns: methodology and applications with R.

[20] Wickham H (2009). ggplot2: elegant graphics for data analysis.

[21] Subramanian A, Tamayo P, Mootha VK, Mukherjee S, Ebert BL, Gillette MA (2005). Gene set enrichment analysis: a knowledge-based approach for interpreting genome-wide expression profiles. Proc Natl Acad Sci U S A.

[22] Arribas AJ, Campos-Martín Y, Gómez-Abad C, Algara P, Sánchez-Beato M, Rodriguez-Pinilla MS (2012). Nodal marginal zone lymphoma: gene expression and miRNA profiling identify diagnostic markers and potential therapeutic targets. Blood.

[23] Yang Z-Z, Grote DM, Ziesmer SC, Xiu B, Novak AJ, Ansell SM (2015). PD-1 expression defines two distinct T-cell sub-populations in follicular lymphoma that differentially impact patient survival. Blood Cancer J.

[24] Rao DA, Gurish MF, Marshall JL, Slowikowski K, Fonseka CY, Liu Y (2017). Pathologically expanded peripheral T helper cell subset drives B cells in rheumatoid arthritis. Nature.

[25] Maley CC, Koelble K, Natrajan R, Aktipis A, Yuan Y (2015). An ecological measure of immune-cancer colocalization as a prognostic factor for breast cancer. Breast Cancer Res.

[26] Lee AM, Clear AJ, Calaminici M, Davies AJ, Jordan S, MacDougall F (2006). Number of CD4+ cells and location of forkhead box protein P3-positive cells in diagnostic follicular lymphoma tissue microarrays correlates with outcome. J Clin Oncol.

[27] Linterman MA, Beaton L, Yu D, Ramiscal RR, Srivastava M, Hogan JJ (2010). IL-21 acts directly on B cells to regulate Bcl-6 expression and germinal center responses. J Exp Med.

[28] Linterman MA, Vinuesa CG (2010). Signals that influence T follicular helper cell differentiation and function. Semin Immunopathol.

[29] Pangault C, Amé-Thomas P, Ruminy P, Rossille D, Caron G, Baia M, De Vos J, Roussel M, Monvoisin C, Lamy T, Tilly H, Gaulard P, Tarte K, Fest T (2010). Follicular lymphoma cell niche: identification of a preeminent IL-4-dependent TFH–B cell axis. Leukemia.

[30] Amé-Thomas P, Le Priol J, Yssel H, Caron G, Pangault C, Jean R (2012). Characterization of intratumoral follicular helper T cells in follicular lymphoma: role in the survival of malignant B cells. Leukemia.

[31] Clipson A, Wang M, De Leval L, Ashton-Key M, Wotherspoon A, Vassiliou G (2015). KLF2 mutation is the most frequent somatic change in splenic marginal zone lymphoma and identifies a subset with distinct genotype. Leukemia.

